# Body Mass Index May Influence Heart Rate Variability

**DOI:** 10.5935/abc.20180201

**Published:** 2018-10

**Authors:** Thalys Sampaio Rodrigues, Levindo José Garcia Quarto

**Affiliations:** 1 University of Melbourne Department of Medicine Austin Health, Heidelberg, Victoria - Austrália; 2 Hospital Regional Norte, Sobral, CE - Brazil

**Keywords:** Hypertension/prevalence, Diabetes Mellitus, Type 2, Risk Factors, Cardiovascular Diseases, Autonomic Nervous Systems, Heart Rate

We read with interest the article by Bassi et al.,^1^ titled “Effects of Coexistence Hypertension and Type II Diabetes on Heart Rate Variability and Cardiorespiratory Fitness”, published in the issue of July 2018. The authors investigated the influence of systemic hypertension on cardiac autonomic modulation in patients with type 2 diabetes mellitus (T2DM) and assessed the heart rate variability (HRV) on exercise capacity in these patients. They concluded that hypertension negatively affects cardiac autonomic function, with greater impairment in HRV, when compared to normotensive patients with T2DM.

Several aspects of this study require discussion. As previously reported, numerous factors may have impact on HRV indices, including sex, insulin resistance, body mass index (BMI), hyperlipidemia, hypertension, ischemic and non-ischemic cardiomyopathy, and smoking status.^2^^-^^4^ For instance, increased BMI can independently decrease HRV, particularly when central adiposity is present.^5^ Indeed, the hypertensive group had a higher BMI when compared to the normotensive group (28 ± 4.4 vs 31 ± 3.8, p = 0.031). Given the lack of control for BMI between the two groups, the conclusions made by the authors should be regarded cautiously.

Finally, it is known that subclinical myocardial dysfunction is highly prevalent in diabetic patients and is independently associated with cardiac autonomic neuropathy.^6^ However, the authors only considered medical history consistent with ischemic heart disease for stratification/exclusion of patients for analysis. We believe that a more detailed cardiovascular assessment, including echocardiography to determine left ventricular mass and left ventricular diastolic dysfunction, would be important to better stratify the patients and strengthen their conclusions.

## References

[1] Bassi D, Cabiddu R, Mendes RG, Tossini R, Arakilian VM, Caruso FC (2018). Effects of coexistence hypertension and type II diabetes on heart
rate variability and cardiorespiratory fitness. Arq Bras *Cardiol*.

[2] Benichou T, Pereira B, Mermillod M, Tauveron I, Pfabigan D, Magdasy S (2018). Heart rate variability in type 2 diabetes mellitus: A systematic
review and meta-analysis. PloSone.

[3] Liao D, Sloan RP, Cascio WE, Folsom AR, Liese AD, Evans GW (1998). Multiple metabolic syndrome is associated with lower heart rate
variability: the Atherosclerosis Risk in Communities Study. Diabetes Care.

[4] Vasconcelos DF, Junqueira Junior LF (2012). Cardiac autonomic and ventricular mechanical functions in
asymptomatic chronic chagasic cardiomyopathy. Arq Bras Cardiol.

[5] Windham BG, Fumagalli S, Ble A, Sollers JJ, Thayer JF, Najjar SS (2012). The relationship between heart rate variability and adiposity
differs for central and overall adiposity. J Obes.

[6] Sacre JW, Franjic B, Jellis CL, Jenkins C, Coombes JS, Marwick TH (2010). Association of cardiac autonomic neuropathy with subclinical
myocardial dysfunction in type 2 diabetes. JACC: Cardiovasc Imaging.

