# Supplementary material for: Body Mass Index May Influence Heart Rate Variability
Source: Arq Bras Cardiol. 2018 Oct;111(4):640–2. doi: 10.5935/abc.20180201 (PMC6199506; doi:10.5935/abc.20180201)
Supplement: Related texts

# Reply

**Authors:** Daniela Bassi, Ramona Cabiddu, Audrey Borghi-Silva

**Dear Editor,**

We appreciate the authors’ interest towards our article, “Effects of Coexistence Hypertension and Type II Diabetes on Heart Rate Variability and Cardiorespiratory Fitness”. We also appreciate the opportunity to respond to their comments. Their critique of our study mainly focused on 3 issues: 1) lack of methodological attention in relation to the participants’ gender, BMI and insulin resistance; 2) lack of methodological details about the study population, regarding smoking habits and hyperlipidemia; and 3) lack of consistent investigation on ischemic and non-ischemic cardiomyopathy. We appreciate the authors’ concerns; however, we do not agree with many of their comments.

The first issue was clearly acknowledged in our paper. Since cardiac variability dynamics differ between genders, with higher parasympathetic activity and overall complexity for women, gender distribution must be considered when investigating heart rate dynamics.^1^ However, in our study, gender distribution is not significantly different for the investigated groups (Diabetes Mellitus (DM) and DM + Hypertension Systemic arterial hypertension (SAH), p = 0.464). In relation to insulin resistance, we agree with the authors’ comments about differences possibly influencing HRV indices. However, we believe that the difference found between groups could be attributed to different weight and BMI. We kindly invite the authors to read a recent study from our group, demonstrating that obesity *per se* impairs aerobic-hemodynamic responses to exercise but that, however, metabolic syndrome (obesity, DM and hypertension) in young adults negatively impacts overall HRV, parasympathetic activity and HRV complexity, corroborating our findings.^2^ In the present study, differences were observed between groups for BMI and weight, with patients in the DM group being overweight and patients in the DM + Hypertension group presenting grade 1 obesity; however, we would like to emphasize that, after age, sex and BMI adjustments, we concluded that these variables did not influence our results.

As for the second issue, current smokers were excluded from our study, as previous evidence showed that tobacco use represents an important cardiovascular risk and leads to HRV impairment.^3^ Even though it was not detailed in the exclusion criteria section, Table 1 clearly shows that none of the participants were current smokers. As for dyslipidemia, we agree with the authors that it may influence HRV; however, after adjustments for this variable, we concluded that dyslipidemia did not significantly influence our results (p = 1.000).

The last issue is related to the lack of consistent investigation on ischemic and non-ischemic cardiomyopathy in the present study. The authors have criticized that a simple clinical evaluation may not be sufficient to determine the presence of ischemic conditions; however, clinical investigation may indicate the need for further exams, aimed at detecting ischemic and non-ischemic heart disease. In addition, the absence of effort-induced ischemic signs was evident during the cardiopulmonary test. Even though this was not clearly stated in the text, we emphasized that all participants underwent a thorough clinical evaluation, consisting of physical examination, resting electrocardiogram and maximal incremental exercise. We kindly invite the authors to read a relevant study about screening procedures for this kind of patients.^4^ Our patients presented no signs or symptoms of suspected ischemic disease, neither at rest, nor during effort. Thus, according to the most recent guidelines for investigation of ischemic patients with DM,^5^ no further examination was needed through echocardiography or other exams to investigate the presence of myocardial dysfunction. 

Finally, it is well known that hypertension *per se* negatively affects HRV;^6^ however, no previous studies investigated linear and non-linear HRV indices in DM + SAH coexistence. Thus, we believe that our article provides a relevant contribution to the understanding of HRV alterations in pathological conditions.

In consideration of the fact that HRV is highly influenced by a number of variables, including demographic and anthropometric characteristics, the presence of obesity, associated comorbidities and cardiovascular risk factors, future, robust studies are needed to further investigate the influence of specific variables on linear and non-linear HRV indices, in order to confirm the preliminary findings of our study.

## References

[1] Ryan SM, Goldberger AL, Pincus SM, Mietus J, Lipsitz LA (1994). Gender- and age-related differences in heart rate dynamics:
are women more complex than men?. J Am Coll Cardiol.

[2] Carvalho LP, Di Thommazo-Luporini L, Mendes RG, Cabiddu R, Ricci PA, Basso-Vanelli RP (2018). Metabolic syndrome impact on cardiac autonomic modulation and
exercise capacity in obese adults. Auton Neurosci.

[3] Barutcu I, Esen AM, Kaya D, Turkmen M, Karakaya O, Melek M (2005). Cigarette smoking and heart rate variability: dynamic
influence of parasympathetic and sympathetic maneuvers. Ann Noninvasive Electrocardiol.

[4] Young LH, Wackers FJT, Chyun DA, Davey JA, Barrett EJ, Taillefer R (2009). Cardiac outcomes after screening for asymptomatic coronary
artery disease in patients with type 2 diabetes. JAMA.

[5] Sociedade Brasileira de Diabetes (2017). Diretrizes Sociedade Brasileira de Diabetes 2017-2018.

[6] Lutfi MF, Sukkar MY (2011). The effect of gender on heart rate variability in asthmatic
and normal healthy adults. Int J Health Sci (Qassim).
